# Identification of Symptomatic Fetuses Infected with Cytomegalovirus Using Amniotic Fluid Peptide Biomarkers

**DOI:** 10.1371/journal.ppat.1005395

**Published:** 2016-01-25

**Authors:** Cyrille Desveaux, Julie Klein, Marianne Leruez-Ville, Adela Ramirez-Torres, Chrystelle Lacroix, Benjamin Breuil, Carine Froment, Jean-Loup Bascands, Joost P. Schanstra, Yves Ville

**Affiliations:** 1 Department of Obstetrics and Fetal Medicine, Hospital Necker-Enfants-Malade, APHP, Paris, France; 2 Institut National de la Santé et de la Recherche Médicale (INSERM), U1048, Institute of Cardiovascular and Metabolic Disease, Toulouse, France; 3 Université Toulouse III Paul-Sabatier, Toulouse, France; 4 Department of Virology, Hospital Necker-Enfants Malades, AP-HP, Paris, France; 5 University Paris Descartes, EA 7328, Paris, France; 6 Sanford Burnham Prebys Medical Discovery Institute, La Jolla, California, United States of America; 7 Centre National de la Recherche Scientifique, Institut de Pharmacologie et de Biologie Structurale, Toulouse, France; Duke University, UNITED STATES

## Abstract

Cytomegalovirus (CMV) is the most common cause of congenital infection, and is a major cause of sensorineural hearing loss and neurological disabilities. Evaluating the risk for a CMV infected fetus to develop severe clinical symptoms after birth is crucial to provide appropriate guidance to pregnant women who might have to consider termination of pregnancy or experimental prenatal medical therapies. However, establishing the prognosis before birth remains a challenge. This evaluation is currently based upon fetal imaging and fetal biological parameters, but the positive and negative predictive values of these parameters are not optimal, leaving room for the development of new prognostic factors. Here, we compared the amniotic fluid peptidome between asymptomatic fetuses who were born as asymptomatic neonates and symptomatic fetuses who were either terminated in view of severe cerebral lesions or born as severely symptomatic neonates. This comparison allowed us to identify a 34-peptide classifier in a discovery cohort of 13 symptomatic and 13 asymptomatic neonates. This classifier further yielded 89% sensitivity, 75% specificity and an area under the curve of 0.90 to segregate 9 severely symptomatic from 12 asymptomatic neonates in a validation cohort, showing an overall better performance than that of classical fetal laboratory parameters. Pathway analysis of the 34 peptides underlined the role of viral entry in fetuses with severe brain disease as well as the potential importance of both beta-2-microglobulin and adiponectin to protect the injured fetal brain infected with CMV. The results also suggested the mechanistic implication of the T calcium channel alpha-1G (CACNA1G) protein in the development of seizures in severely CMV infected children. These results open a new field for potential therapeutic options. In conclusion, this study demonstrates that amniotic fluid peptidome analysis can effectively predict the severity of congenital CMV infection. This peptidomic classifier may therefore be used in clinical settings during pregnancy to improve prenatal counseling.

## Author Summary

CMV is the most common cause of congenital infection, and can result in significant neonatal morbidity and neurological disabilities. The birth prevalence of congenital CMV is estimated at 0.7% worldwide, and 10 to 20% of these neonates develop severe symptoms. In such cases the outcome is generally poor. Therefore, identification of additional prognostic markers is crucial for prenatal counseling in cases with an infected fetus. This may influence the decision of continuing with the pregnancy or requesting its termination, but also the decision of starting experimental antiviral therapy. The pathophysiology of CMV brain injury is not completely understood, and the identification of new biomarkers of CMV infection might also pave the way towards the development of new therapeutic alternatives. Here, we apply a recently developed and modern non-targeted peptidomics approach to amniotic fluid obtained from symptomatic and asymptomatic CMV-infected fetuses/neonates, followed by network analysis of the peptides of interest in the context of fetal infection and in relation with outcome. Our study identified 34 amniotic fluid peptides that form new prognostic biomarkers that could be used in clinical settings to improve prenatal counseling. In addition, this study provides novel mechanistic insight into the pathobiology of CMV congenital disease.

## Introduction

Cytomegalovirus (CMV) is the most common cause of congenital infection with an incidence of 0.7% at birth [1]. Congenital CMV infection is the leading cause of non-genetic hearing loss and the most frequent viral cause of neurodevelopmental delay. Primary maternal CMV infection in pregnancy carries a 30% to 40% risk of vertical transplacental transmission, and 10% of those infected fetuses will be born as infected infants with clinical symptoms and long-term disabilities including sensorineural hearing loss and cognitive deficits such as mental retardation, cerebral palsy or seizures [2]. In addition, 5 to 10% of asymptomatic infants will develop milder forms of sensorineural hearing loss and of psychomotor delay later in life [2].

When maternal primary infection is documented, it is important to evaluate the risk to the fetus of being infected and/ symptomatically affected by CMV in order to provide appropriate counseling to these pregnant women [3]. Fetal CMV infection is diagnosed by amplification of the viral DNA in the amniotic fluid obtained by amniocentesis. However, a diagnosis of fetal CMV infection does not equate to a symptomatic neonate since 80% to 90% of fetuses with congenital CMV infection are asymptomatic at birth. Prenatal assessment of prognosis is mainly based upon ultrasound or MRI imaging of the fetal brain or fetal blood biochemistry. When severe ultrasound features are present at the time of diagnosis in the second trimester of pregnancy, the prognosis is known to be poor [4][5]. However, when the infected fetus shows either no or only non-severe ultrasound anomalies, the prognosis is difficult to establish. Indeed, although the negative predictive value of fetal imaging has been reported to be as high as 90% [6,7], damages to the fetal brain may be delayed or masked to ultrasound, and even to magnetic resonance imaging (MRI), up until late in the third trimester or at birth. Fetal blood sampling by cordocentesis under ultrasound guidance has been advocated for the additional prognostic value of fetal platelet count, CMV DNA levels and beta-2-microglobulin serum levels [8,9]. However, this procedure is invasive and is associated with a 1–3% risk of fetal loss. Finally, the prognostic value of the levels of CMV DNA loads in amniotic fluid is controversial [10–13]. Altogether, the prenatal prognosis of fetal CMV infection remains difficult to establish. In the past, the only option discussed when infected fetuses were suspected of severe disabilities was elective termination of pregnancy (TOP). Alternative medical strategies such as CMV hyperimmune globulins and other antiviral drugs are being evaluated with recent studies showing conflicting results on the incidence of sequelae among treated infected fetuses [14][15]. Therefore, the identification of new effective prognostic markers is critical to help counseling pregnant women carrying an infected fetus through the dilemma of continuing or not with their pregnancy but also to decide upon starting medical therapy *in utero*.

Amniotic fluid has been subjected to proteome analysis (*i*.*e*. analysis of the global protein content of a sample) for the identification of biomarkers of several prenatal conditions [16]. Specific amniotic fluid proteins have been related to various conditions affecting the fetus including Down-syndrome [17][18], intrauterine inflammation leading to preterm birth [19][20], preterm premature rupture of the membranes [21] and intrauterine growth restriction [22]. Moreover, we and others have shown that capillary electrophoresis coupled to mass spectrometry (CE-MS) analysis of body fluids can help identifying peptide-based biomarkers of disease which can be clinically relevant [23–25].

In this study, our aim was to analyze the amniotic fluid peptidome of CMV infected fetuses at mid-gestation and to evaluate the presence of peptide biomarkers that could contribute to improve the prognostic evaluation of this condition.

## Results

### Identification of amniotic fluid peptides associated with the severity of postnatal outcome

**Fig 1:**
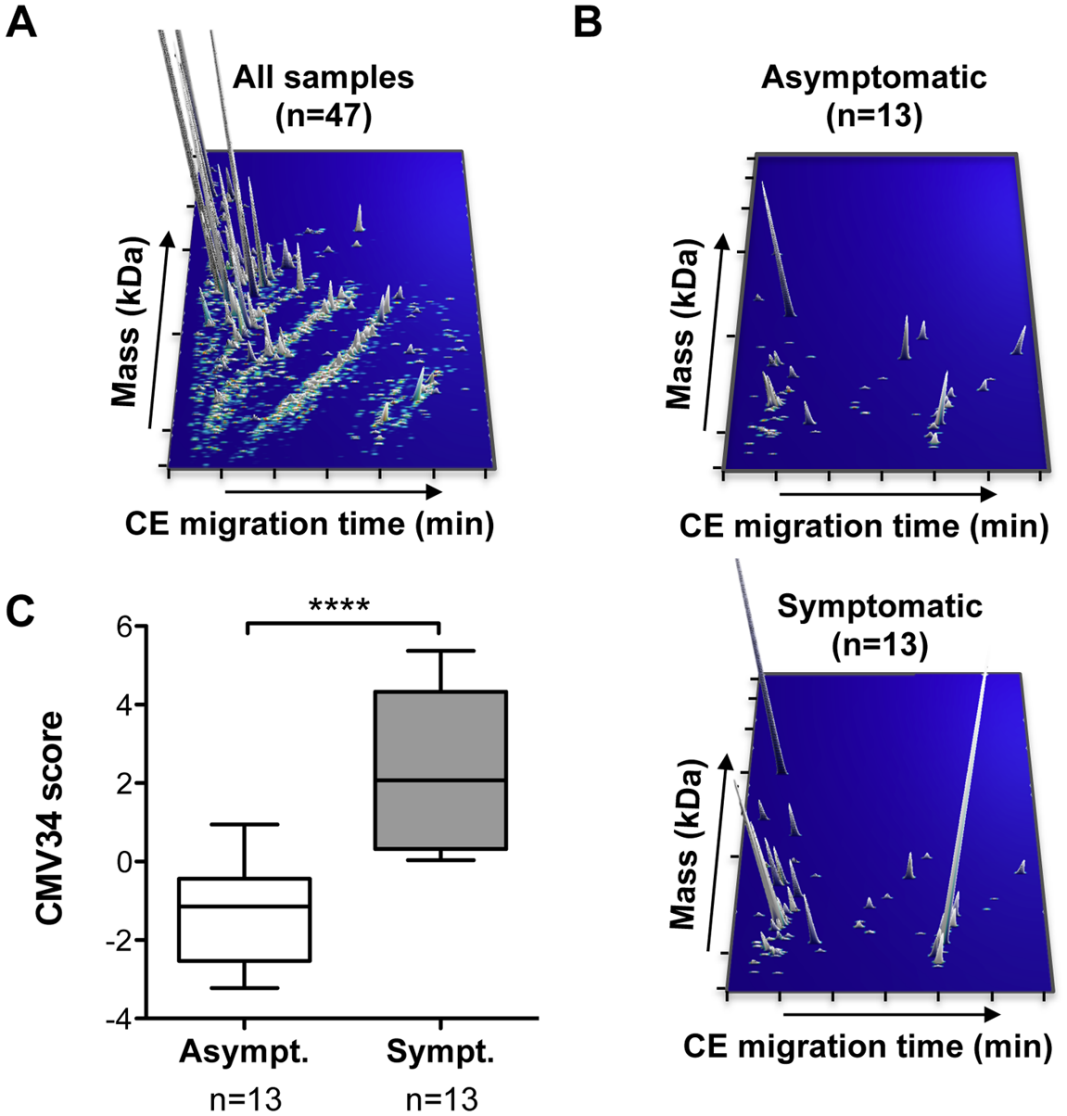
Detected peptides in amniotic fluid and differences between symptomatic and asymptomatic cases in the discovery cohort. (**A**) Representation of 4076 peptides, detected in all amniotic fluid samples (n = 47) by CE-MS. Each peptide was identified by a unique identifier based on the migration time (min) and specific mass (kDa), with a peak height representing the relative abundance. (**B**) In the discovery cohort, 76 amniotic fluid peptides were identified as differentially secreted between symptomatic and asymptomatic patients. (**C**) Cross-validation score of a SVM peptide classifier called CMV34 consisting of 34 of the 37 sequenced peptides obtained from the analysis of the discovery cohort. ***p<0.0001, Mann-Whitney test for independent samples. Asympt., asymptomatic; Sympt., symptomatic.

CE-MS-based peptidome analysis using 150 μL of amniotic fluid from 47 samples from CMV infected fetuses allowed the detection of a total of 4076 different peptides in all samples (Fig 1A). The samples were further divided in a discovery and validation cohorts (Table 1). The discovery cohort consisted of 26 pregnancies with primary CMV infection including 13 severely symptomatic cases (Table 1) and 13 asymptomatic cases. All amniotic fluid samples were collected between 17 and 29 weeks of gestation with a mean of 22 weeks of gestation in the asymptomatic group and of 23 weeks in the severely symptomatic group (p = 0.43, not significant) (Table 1 and S1 Table). Follow-up of asymptomatic infants was obtained up to 12 to 53 (mean 24) months of age. In the severely symptomatic group, 12 fetuses underwent TOP for severe brain lesions and all but one were confirmed at necropsy (Table 2). One child was born alive but developed as severely handicapped (Table 2). Comparison of the amniotic fluid peptides content of the discovery cohort led to the identification of 76 peptides that were consistently differentially excreted between severely symptomatic and asymptomatic cases between these two groups (Fig 1B, S1 and S2 Datasets). These 76 amniotic fluid peptides are identified by a unique tag composed of specific mass and migration time. In the next step, using a combination of LC-MS/MS and CE-MS/MS, sequence information could be obtained in 37 of these 76 peptides. Most of these peptides were fragments of various collagens. However, a number of other non collagen-related peptides (13 out of 37) were also observed, including a fragment of hemoglobin subunit delta and of beta-2-microglobulin displaying a strong increase in abundance in amniotic fluid of symptomatic patients (>8-fold change)(Table 3). The 37 sequenced peptides were reduced to a support vector machine (SVM) classifier with 34 peptides, called “CMV34”, by a leave-one-out procedure whereby the classifier was assessed for all peptide candidates minus one. Peptides that did not influence the accuracy of the classifier in the total cross-validation of the training data were left out of the final classifier. Scoring the patients from the discovery cohort with the resulting CMV34 classifier clearly separated the majority of the symptomatic from asymptomatic patients (Fig 1C).

**Table 1:** Patient characteristics.

	Discovery cohort		Validation cohort		
	Asympt.	Sympt.	Asympt.	Sympt.	Hearing loss and vestibular dysfunction
**Number**	13	13	12	9	9
**Gestational age at sampling (weeks of gestation)**	22.4 ±0.9	23.0 ±0.5	24.7 ±1.4	23.4 ±1.0	22.6 ±0.9
**CMV DNA levels in amniotic fluid (log10 copies/mL)**	5.4 ±0.9	7.4 ±0.3**	5.8 ±0.3	7.2 ±0.5*	7.3 ±0.4
**CMV DNA levels in fetal blood (log10 copies/mL)**	4.2 ±0.3	5.2 ±0.2*	4.3 ±0.3	5.6 ±0.2*	4.9 ±0.2

*p<0.05 and **p<0.01 compared to asymptomatic group, Mann-Whitney test for independent samples.

Data are presented as mean±SEM. Asymt.: asymtomatic; Sympt.: symptomatic.

**Table 2:** Prenatal ultrasound data, outcome and postnatal examination of the severely symptomatic patients from the discovery cohort.

Patient ID	Prenatal ultrasound data	MRI	Outcome	Postnatal examination
**DC_S14**	Hyperechogenic bowel, ventriculomegaly measured at 11mm	Microcephaly, white matter lesions	TOP (32w)	Microcephaly, ventriculomegaly, periventricular leukomalacia, hepatomegaly, hepatocellular necrosis, tubulointerstitial nephritis
**DC_S15**	Hyperechogenic bowel, placentomegaly, microcephaly, periventricular necrosis, lateral ventricle partitioning	Not done	TOP (24w)	Microencephaly, Periventricular leukomalacia, hydrocephaly, micropolygyria, hepatitis, placental vilitis
**DC_S16**	IUGR, hyperechogenic bowel, multiple intracerebral calcifications	Not done	TOP (25w)	Hydrocephalus, left hemisphere atrophy, diffused gliosis basal ganglia calcifications and vasculitis
**DC_S17**	Microcephaly, growth restriction, hepatomegaly, hyperechogenic bowel, multiple intra cranial calcification, hypoplasia of the corpus callosum	Not done	TOP (26w)	Hydrocephalus, diffused gliosis, micropolygyria, cerebral vasculitis hypotrophy and meningitis
**DC_S18**	IUGR, microcephaly, multiple intra cerebral calcifications, ventriculomegaly	Microencephaly, white matter lesions	Birth	Severe psychomotor developmental delay, epilepsy, bilateral severe hearing loss, diagnosed at birth and at the age of 8
**DC_S19**	Ventriculomegaly, lissencephaly, intra cerebral clastic lesions	Lissencephaly, polymicrogyria, white matter lesions	TOP (33w)	Hydrocephalus, periventricular leukomalacia, polymicrogyria, hepatomegaly, splenomegaly
**DC_S20**	Hepatomegaly, ascites, hyperechogenic bowel, ventriculomegaly, periventricular leukomalacia	Not done	TOP (24w)	Microcephaly, periventricular leukomalacia, cerebellar necrosis, diffuse white matter necrosis, hepatomegaly, splenomegaly, liver necrosis
**DC_S21**	Ventriculomegaly measured at 11mm, brain gyration abnormalities, multiple intracranial calcifications	Not done	TOP (25w)	Polymicrogyria, diffuse white matter necrosis and gliosis, chronic placental vilitis
**DC_S22**	Liver calcifications, hyperechogenic bowel, microcephaly, multiple intra cerebral calcifications	Not done	TOP (27w)	Microcephaly multiple intra cerebral calcifications and gliosis
**DC_S23**	Lateral ventricle partitioning,	Diffuse white matter lesions, intraventricular adhesions.	TOP (28w)	Encephalitis, white matter necrosis, chronic placental vilitis
**DC_S24**	Hyperechogenic bowel	Not done	TOP (23w)	Grade 4 Intra ventricular hemorrhage, diffuse gliosis, micro-encephaly, placental villitis
**DC_S25**	Microcephaly, ventriculomegaly, hyperechogenic bowel, liver calcifications, IUGR	Not done	TOP (24w)	Microcephaly, hydrocephalus, grade 3 intraventricular hemorrhage, exoensive white matter necrosis, diffuse intra cerebral calcifications, liver necrosis
**DC_S26**	Ascitis, pericardial effusion, hydrocephalus	Not done	TOP (25w)	Not done

TOP, termination of pregnancy; IUGR, intra uterine growth restriction; w, weeks of pregnancy

**Table 3:** Peptides used in the CMV34 classifier.

Peptide ID	Parent protein (symbol)	Parent protein (name)	Fold change S/A	Direction S/A
**7094**	VRK3	Inactive serine/threonine-protein kinase VRK3	2.4	Up
**15776**	COL2A1	Collagen alpha-1(II) chain	3.4	Up
**15800**	CACNA1G	T calcium channel alpha 1G subunit variant 249	2.3	Up
**17901**	FAM135B	Protein FAM135B	2.8	Up
**18077**	ZC3H6	Zinc finger CCCH domain-containing protein 6	4.2	Up
**18943**	COL3A1	Collagen alpha-1(III) chain	2	Up
**19597**	SCRIB	Protein scribble homolog	2.4	Up
**22089**	MCM6	DNA replication licensing factor MCM6	2	Up
**24502**	CCDC66	Coiled-coil domain-containing protein 66	2	Up
**26896**	HBD	Hemoglobin subunit delta	8.6	Up
**27350**	COL1A1	Collagen alpha-1(I) chain	3.3	Up
**28466**	TSC2	Tuberin	3.2	Up
**34795**	ADIPOQ	Adiponectin	3.8	Up
**35339**	COL1A1	Collagen alpha-1(I) chain	10.6	Up
**36858**	COL19A1	Collagen alpha-1(XIX) chain	2.9	Up
**37056**	PIK3CB	Phosphatidylinositol 4;5-bisphosphate 3-kinase catalytic subunit beta isoform	2.9	Up
**37662**	COL25A1	Collagen alpha-1(XXV) chain	3.5	Up
**41770**	COL16A1	Collagen alpha-1(XVI) chain	5.6	Up
**41833**	COL16A1	Collagen alpha-1(XVI) chain	5.1	Up
**44679**	B2M	Beta-2-microglobulin form pI 5.3	11.9	Up
**49295**	COL3A1	Collagen alpha-1(III) chain	4.4	Up
**50338**	COL1A2	Collagen alpha-2(I) chain	0.01	Down
**51929**	PRKD1	Serine/threonine-protein kinase D1	3.3	Up
**58142**	COL23A1	Collagen alpha-1(XXIII) chain	0.2	Down
**59369**	COL1A1	Collagen alpha-1(I) chain	0.2	Down
**59923**	COL3A1	Collagen alpha-1(III) chain	3.4	Up
**61332**	COL3A1	Collagen alpha-1(III) chain	3	Up
**71171**	COL3A1	Collagen alpha-1(III) chain	3.9	Up
**72596**	COL1A1	Collagen alpha-1(I) chain	2.7	Up
**78332**	COL1A1	Collagen alpha-1(I) chain	0.5	Down
**79136**	COL1A1	Collagen alpha-1(I) chain	0.6	Down
**84484**	COL4A2	Collagen alpha-2(IV) chain	0.5	Down
**95746**	COL3A1	Collagen alpha-1(III) chain	5.4	Up
**127432**	COL1A1	Collagen alpha-1(I) chain	0.3	Down

S/A, symptomatic/asymptomatic.

### Validation of the multidimensional amniotic fluid peptide classifier in a blinded validation cohort

In the next step the CMV34 classifier was validated in a separate blinded cohort using 21 amniotic fluid samples of primary CMV infection pregnancies. Amniocentesis was performed between 18 and 30 weeks of gestation with means of 25 and 23 weeks in the asymptomatic and in the severely symptomatic groups respectively (p = 0.49, not significant)(Table 1 and S1 Table). The cohort consisted of 9 severely symptomatic and 12 asymptomatic fetuses that were not used in the discovery phase (Table 1). In the severely symptomatic group, all cases underwent TOP for severe brain lesions (Table 4). Six out of the 9 symptomatic fetuses had an autopsy and all confirmed the severity of the lesions. In the other 3 cases, although autopsy was not performed, prenatal imaging described indisputably severe cerebral lesions on ultrasound that correlate well with all other cases terminated on the same basis and confirmed by postmortem examination. We have therefore no reason to believe that such a rich cerebral semeiology could be a false positive interpretation. The mean postnatal follow-up of the asymptomatic group was 8 months.

**Table 4:** Prenatal ultrasound data, outcome and postnatal examination of the severely symptomatic patients from the validation cohort.

Patient ID	Prenatal ultrasound data	MRI	Outcome	Postnatal examination
**VC_S13**	Hyperechogenic bowel, placentomegaly, microcephaly, periventricular necrosis, lateral ventricle partitioning	Not done	TOP (25 W)	Microcephaly, periventricular necrosis, diffuse gliosis, white matter necrosis, intraventricular adhesions, hepatomegaly, splenomegaly, liver necrosis
**VC_S14**	IUGR, hyperechogenic bowel, microcephaly	Microcephaly, white matter lesions	TOP (28W)	Microcephaly, microencephaly, chronic placental vilitis
**VC_S15**	Ventriculomegaly, hyperechogenic bowel, cystic periventricular leukomalacia	Not done	TOP (25w)	Not done
**VC_S16**	Oligohydramnios, ascites, microcephaly, abnormal gyration, candlestick	Microcephaly, lissencephaly, white matter lesion	TOP (28 W)	Not done
**VC_S17**	Hydrocephalus, microcephaly, severe white matter lesions	Not done	TOP (25 w)	Not done
**VC_S18**	Microcephaly, IUGR, cardiomegaly, splenomegaly, oligohydramnios, ventriculomegaly, periventricular calcifications	Microcepahly, ventriculomegaly	TOP (29 W)	Hydrocephalus, microcephaly, diffuse gliosis and necrosis, multiple intra cerebral calcifications, placental villitis, IUGR
**VC_S19**	IUGR <10°P, microcephaly, hyperechogenic bowel, splenomegaly	Not done	TOP (25 W)	Microcephaly, diffuse gliosis, hepatomegaly, splenomegaly
**VC_S20**	IUGR, lateral ventricle partitioning, microcephaly	Microcephaly, lissencephalie, ventriculomegaly	TOP (27 W)	IUGR, polymicrogyria, microcephaly diffuse gliosis and necrosis of the white matte
**VC_S21**	Hydrocephalus, Microcephaly, intraventricular hemorrhage, ascites, pleural effusion, anemia	Not done	TOP (19 W)	IUGR, microcephaly, meningoencephalitis, brain hypotrophy and necrosis, diffuse gliosis

TOP, termination of pregnancy; IUGR, intra uterine growth restriction; w, weeks of pregnancy

**Fig 2:**
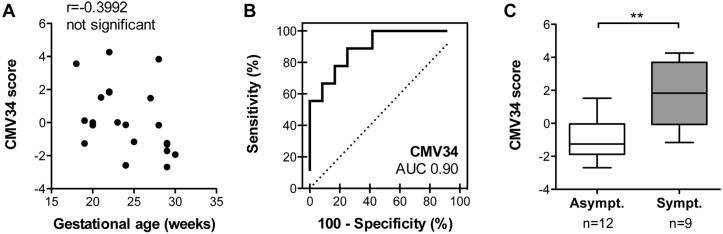
Performance of the CMV34 classifier in the validation cohort. (**A**) Correlation analysis of the CMV34 classifier and gestational age. (**B**) ROC curve for the CMV34 classifier. (**C**) Box-whisker plot for classification of symptomatic and asymptomatic patients in the validation set according to the CMV34 score. **p<0.01, Mann-Whitney test for independent samples.

Amniotic fluid samples were analyzed by CE-MS (S3 and S4 Datasets), scored using the CMV34 classifier and then compared with the clinical data. We first verified that the CMV34 classifier score was independent of gestational age (Spearman r = -0.3992, not significant) (Fig 2A). The CMV34 classifier predicted a symptomatic/asymptomatic outcome with 89% sensitivity and 75% specificity with an area under the curve (AUC) of 0.90 [95% CI: 0.68 to 0.98] (Fig 2B and Table 5). The distribution of CMV34 scores for the validation cohort also showed highly significant separation of the 2 populations (p = 0.0025, Fig 2C). Among the 12 asymptomatic fetuses, 3 were wrongly classified as symptomatic by the CMV34 classifier when considering a score above/below 0 as threshold (VC_AS2, 1.523; VC_AS6, 0.122; VC_AS8, 0.004 (S1 Table). Imaging data showed that for the first misclassified asymptomatic fetus, the only ultrasound symptom was hyperechogenic bowel. MRI done at 32 weeks of gestation did not show any abnormality. The child was asymptomatic at 24 months of age. For the second misclassified fetus, no abnormalities were seen at antenatal ultrasound nor at MRI. The child was still asymptomatic at 8 months of age. The third fetus showed no abnormalities were seen at antenatal ultrasound nor at MRI. The child was asymptomatic at birth and then lost for follow-up. Among the 9 fetuses considered as severely symptomatic, 2 were classified as asymptomatic by the classifier with a score of -0.136 (VC_S16) and of -1.164 (VC_S20) (Table 4 and S1 Table).

**Table 5:** Sensitivity, specificity, AUC, positive predictive value (PPV) and negative predictive value (NPV) of CMV34 and other clinical parameters associated to postnatal outcome.

	Sensitivity (% [95% CI])	Specificity (% [95% CI])	AUC [95% CI]	PPV* (% [95% CI])	NPV* (% [95% CI])
**CMV34** °	89 [51.8–99.7]	75 [42.8–94.5]	0.90 [0.68–0.98]	0.35 [0.03–0.85]	0.98 [0.62–1.00]
**CMV DNA levels in amniotic fluid** °°	79 [54.4–93.9]	84 [63.9–95.5]	0.84 [0.70–0.93]	0.42 [0.05–0.88]	0.96 [0.72–1.00]
**CMV DNA levels in fetal blood** °°	92 [64.0–99.8]	59 [36.4–79.3]	0.81 [0.65–0.92]	0.25 [0.02–0.71]	0.98 [0.55–1.00]
**Fetal platelet count** °°	82 [48.2–97.7]	70 [45.7–88.1]	0.77 [0.58–0.90]	0.29 [0.01–0.83]	0.96 [0.54–1.00]

° Data given for validation cohort only.

°° Data given for both discovery and validation cohort since missing values did not allow a separate analysis of the discovery and validation cohort.

* Based on a prevalence of 13% [1] of symptomatic CMV infected individuals; confidence intervals were calculated using variable numbers of cases: 22 for CMV34; 19 for CMV DNA levels in amniotic fluid, 13 for CMV DNA levels in fetal blood and 11 for fetal platelet count.

### Comparison of the multidimensional amniotic fluid peptide classifier with other clinical parameters

**Fig 3:**
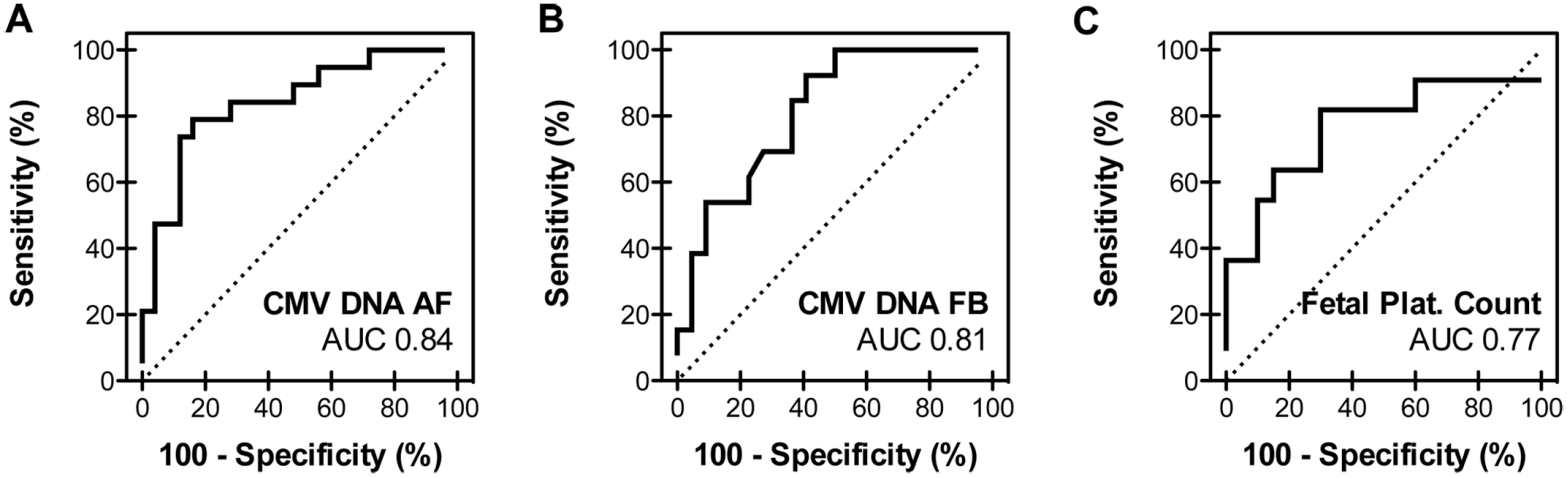
Performance of other frequently used parameters in the validation cohort. ROC curves for CMV DNA levels in amniotic fluid (**A**) and fetal blood (**B**) and the fetal platelet count (**C**) in the combined discovery and validation cohort. AF, amniotic fluid; FB, fetal blood; Plat., platelet.

We next compared the performance of the CMV34 classifier with other frequently used, but controversial [8,9] [10–13], parameters used for the assessment of the severity of the CMV infection including CMV DNA levels in amniotic fluid and fetal blood and the fetal platelet count. Since the number of missing values for these parameters was high, mostly in the symptomatic fetuses (S1 Table) we combined the discovery and validation cohorts. The classifier displayed a higher AUC than the clinical parameters (Fig 3 and Table 5). However, the sensitivity of CMV DNA levels in fetal blood was slightly higher, 92 versus 89% for CMV34 (Table 5). Based on a prevalence of 13% risk of being symptomatic at birth among infected fetuses [1], we also assessed the positive (PPV) and negative (NPV) predictive values. All four parameters displayed high NPV, while the classifier and CMV DNA levels in amniotic fluid showed highest and comparable positive predictive value (Table 5).

### Classification of primary CMV infection with moderately symptomatic neonates

**Fig 4:**
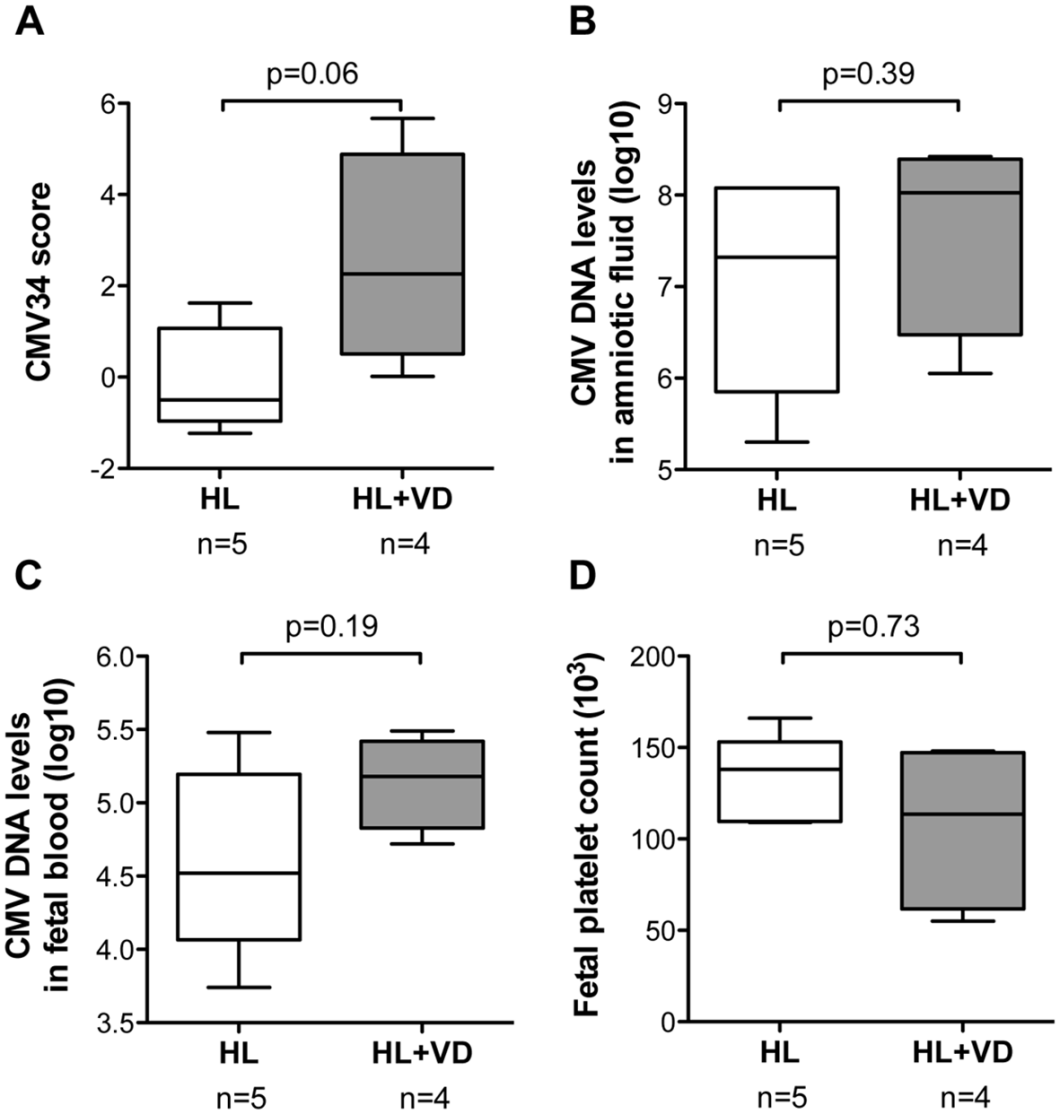
Classification of primary CMV infection with moderately symptomatic neonates. (**A**) The amniotic fluid peptide content of CMV-infected fetuses with moderate neonatal symptoms (hearing loss (HL) or hearing loss and vestibular dysfunction (HL+VD)) was scored with the CMV34 classifier. The scores allowed a nearly significant difference after analysis by Mann-Whitney test for independent samples (p = 0.06). CMV DNA levels in amniotic fluid and fetal blood (**B** and **C**, respectively) and the fetal platelet count (**D**) were clearly not different between hearing loss *versus* hearing loss and vestibular dysfunction (p values of 0.32, 0.19 and 0.73, respectively, Mann-Whitney test for independent samples).

A small group of 9 cases with moderate postnatal symptoms (*i*.*e*. hearing loss and/or vestibular dysfunction) was scored separately using the CMV34 classifier (Table 1 and S2 Table). Amniocentesis was carried out between 19 and 28 (mean 23) weeks. Postnatal follow-up was of 12 to 84 (mean 34) months. All 4 children suffering from vestibular dysfunction and 2 of 5 with isolated hearing loss were classified as symptomatic prenatally by the CMV34 peptide classifier (S2 Table). The comparison of the combined CMV34 scores of these 2 sub-groups (hearing loss and vestibular dysfunction, HL+VD *versus* hearing loss only, HL) showed a trend towards higher scores for children with vestibular dysfunction (p = 0.06)(Fig 4A). Interestingly such trend could not be observed for CMV DNA levels in amniotic fluid and fetal blood and the fetal platelet count (Fig 4B–4D and S2 Table).

### Pathway analysis

**Fig 5:**
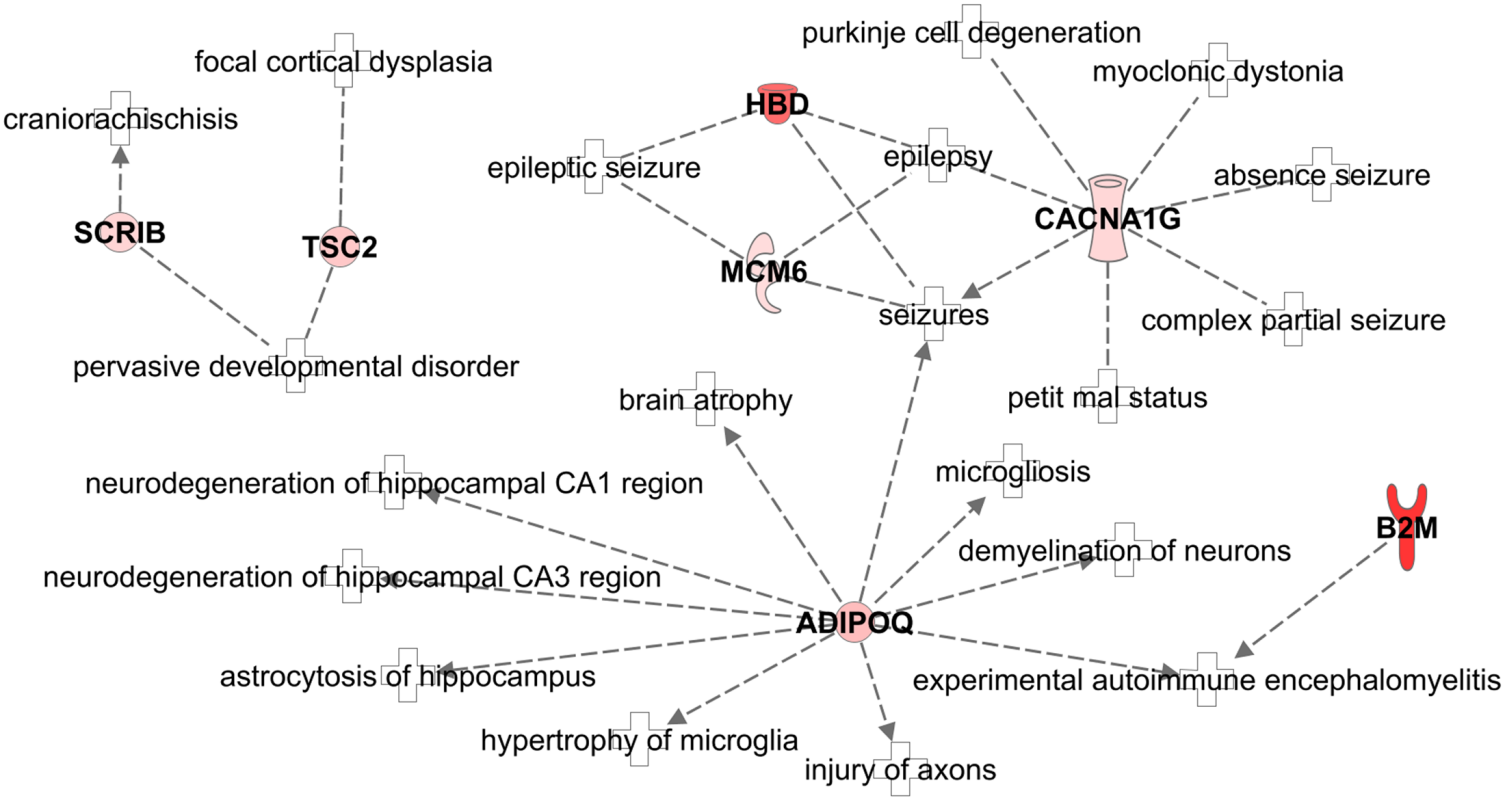
Alteration of the neurological disease pathway. Ingenuity Pathway Analysis software showed a significant activation of neurological disease pathway in symptomatic fetuses compared with asymptomatic, with 7 out of 13 non-collagen peptide parent proteins being associated to the network. Red: increased abundance. B2M, beta-2-microglobulin; HBD, hemoglobin delta subunit; ADIPOQ, adiponectin; SCRIB, protein scribble homolog; CACNA1G, T calcium channel alpha-1G; MCM6, DNA replication licensing factor; TSC2, tuberin.

An advantage of non-targeted approaches leading to panels of peptides is that such data can be subjected to pathway analysis aiming at a deeper understanding of the underlying pathophysiology of the disease. The 13 non-collagen peptides (considering peptides as proteins) were submitted to Ingenuity Pathway Analysis software. The second top canonical pathway (ignoring the first pathway related to HER-2 signaling in breast cancer) was “Virus Entry via Endocytic Pathways” confirming the relation of 3 (beta-2-microglobulin (B2M); Phosphatidylinositol 4;5-bisphosphate 3-kinase (PIK3CB) and protein kinase and Serine/threonine-protein kinase D1 (PRKD1)) out of the 13 peptide markers with the disease under study. When focusing on enrichment of pathways related to diseases, “Organismal Injury and Abnormalities” was among the top pathways suggesting a general connection to the potential lesions observed in symptomatic CMV. Another major enrichment of the peptide biomarkers was found to be in “Neurological Disease” with 7 out of 13 peptides significantly enriched (Fig 5): beta-2-microglobulin (B2M), hemoglobin delta subunit (HBD), adiponectin (ADIPOQ), protein scribble homolog (SCRIB), T calcium channel alpha-1G (CACNA1G), DNA replication licensing factor (MCM6) and tuberin (TSC2). Neurological lesions are the main prognostic lesions among the manifestations observed in symptomatic CMV patients including epilepsy.

## Discussion

The management of fetal CMV infection remains controversial. One difficulty is to establish the prognosis timely and accurately before birth. Prognostic factors are mainly derived from prenatal ultrasound or MRI imaging of the fetal brain and these can either appear late in the pregnancy and/or be objectified only after birth. Laboratory markers of the severity of infection including thrombocytopenia and high serum levels of beta-2-microglobulin in fetal blood have been suggested to precede the development of brain lesions. These can be obtained following cordocentesis, another invasive procedure performed under ultrasound guidance and separate from the amniocentesis performed for diagnosis [8,9,26]. We therefore aimed at identifying reliable protein-based prognostic factors of fetal CMV infection in the amniotic fluid at the time of prenatal diagnosis by amniocentesis.

Amniotic fluid of 26 fetuses infected with CMV contained 76 peptides showing a clearly distinct abundance between those leading to either severely symptomatic or asymptomatic children by the age of 12 months. Thirty-four of the 37 sequenced peptides were combined in a prognostic classifier called CMV34. The classifier was independent of gestational age and reached 89% sensitivity, 75% specificity and an area under the curve of 0.90 to discriminate severely symptomatic from asymptomatic neonates in a validation cohort. The CMV34 classifier showed better AUC and NPV than the other biomarkers of severity of CMV fetal disease including fetal platelets, CMV DNA levels in fetal blood and in amniotic fluid. High NPV, leading to unambiguous identification of asymptomatic neonates, is crucial for clinical management. PPV values were low and comparable for all four biomarkers, which could be expected due to the low prevalence of symptomatic neonates among CMV infected fetuses. Hence, a score suggesting a symptomatic fetus should always to be confirmed by targeted imaging. These data support that the CMV34 classifier may be of substantial value in the clinical context of intrauterine CMV infection.

The selected peptides included 21 collagen fragments suggestive of tissue remodeling differences between symptomatic and asymptomatic patients. The other 13 peptides that were significantly more abundant in amniotic fluid of severely symptomatic fetuses included fragments of beta-2-microglobulin, hemoglobin subunit delta and adiponectin. The beta-2-microglobulin and the hemoglobin subunit delta fragments were highly up-regulated (>8 fold) suggesting an important role of these peptides in congenital CMV disease. Beta-2-microglobulin is the light chain of class I major histocompatibility complex and is present in the majority of T- and B-lymphocytes. Fetal blood levels of beta-2-microglobulin have previously been reported as a prognostic marker of fetal CMV infection [27] and beta-2-microglobulin has also been described as an important antibacterial protein in amniotic fluid [28]. Increased serum levels of beta-2-microglobulin is also a predictive factor of CMV infection in adult renal transplant recipients [29]. In the context of this study, high levels of circulating beta-2-microglobulin might be suggestive of lymphoid cell stimulation. One other hypothesis could be that increased beta-2-microglobulin reflects intense viral replication since it has been shown that CMV binds to beta-2-microglobulin [30]. However, in our study the abundance of the beta-2-microglobulin fragment (ID 44679) was neither correlated to CMV DNA levels in amniotic fluid nor to CMV DNA levels in fetal blood (S1 Fig).

In association with hemoglobin subunit alpha, hemoglobin subunit delta constitutes hemoglobin A2, which represents the adult hemoglobin. The hemoglobin subunit delta peptide found in amniotic fluid is therefore of maternal origin. The high specificity and the large differences between symptomatic and asymptomatic groups (8.6-fold change) led us to hypothesize that those findings are not related to contamination during amniocentesis. Vaibuch et al. have demonstrated that total hemoglobin can be detected in the amniotic fluid of all pregnant women, increasing in concentration with gestational age. They also demonstrated in the same study [31] that the total hemoglobin level in amniotic fluid was significantly higher in women with intra-amniotic infection and/or inflammation. We suggest that this finding of higher hemoglobin subunit delta in amniotic fluid from symptomatic fetuses could be due to a more severe inflammation related to CMV infection.

There is emerging evidence indicating that the study of biological networks can provide insight into the pathobiology of disease and improve biomarker discovery. We used the Ingenuity Pathway Analysis software to identify networks related to the peptides identified within this classifier. The second top canonical pathway was related to virus entry via endocytic pathway. Mechanisms of CMV entry into the cells are not completely understood. There is evidence that CMV enters into fibroblasts using a pathway involving membrane fusion, whereas it enters epithelial and endothelial cells via an endocytic pathway involving macropinocytosis [32]. Up-regulation of the canonical pathways “virus entry” in the more severe cases probably reflects intensive viral multiplication making the case for prenatal antiviral treatment. Moreover, one of the top disease pathways was identified as “neurological” and included 7 proteins all up-regulated in the more severe cases: beta-2-microglobulin (B2M), hemoglobin delta subunit (HBD), adiponectin (ADIPOQ), protein scribble homolog (SCRIB), T calcium channel alpha-1G (CACNA1G), DNA replication licensing factor (MCM6) and tuberin (TSC2).

A previous publication reported elevated level of adiponectin in the amniotic fluid of women with intra-amniotic infection compared to women without infection [33]. Adiponectin appears to be intimately involved in several neurodegenerative disorders that are associated with CMV brain disease, such as epilepsy and ischaemic stroke [34] Therefore, adiponectin up-regulation in severe cases probably underlines the potential importance of inflammatory response on fetal brain lesions; this inflammatory response could be a target for future antenatal therapeutic intervention. The protein scribble homolog (SCRIB) is involved in planar cell polarity and Scrib knockout animal models present with neural tube defects [35]. Up-regulation of SCRIB in fetuses with severely affected brains could be a counter-mechanism of the putative effect of CMV on the differentiation and the migration of neuronal stem cells. Tuberin is the product of gene TSC2 and is highly expressed in fetal neural tissue [36]. Mutation of TSC2 is responsible for the development of Tuberous Sclerosis whereas activation of TSC2 has been reported to increase autophagy of the neurons in a model of Parkinson‘s disease [37]. Finally, T calcium channel alpha-1G (CACNA1G) protein belongs to the neuronal T-type calcium channels that are critical contributors to membrane excitability in neuronal cells. These T-type channels have a role in ischemic neuronal cell death in brain undergoing oxygen-glucose deprivation [38] as could be the case in CMV infection. Up-regulation of T calcium channel alpha-1G protein might thus predict an activation of seizures, which are frequent consequences of severe CMV brain disease. Molecules that counteract the effect of T calcium channel alpha-1G could therefore also be an interesting field of development to help treating the symptoms of children with severe CMV brain disease.

In this study, we did not seek for diagnostic markers of fetal CMV infection but we were investigating prognostic markers in established CMV infected cases. However, it would be interesting to further study whether these markers are specific for CMV infection or could represent, at least partially, global perturbations related to *in utero* infection. Therefore, the CMV34 classifier could be tested on amniotic fluid samples from uninfected fetuses, from other pregnancy-related conditions such as pre-eclampsia, or from other infectious diseases, such as chorioamnionitis.

In conclusion, the analysis of the peptidome in the amniotic fluids infected by CMV led to the identification of a prognostic classifier based on 34 peptides that yielded a better performance than the currently used fetal biomarkers to predict the asymptomatic or symptomatic status at birth. This classifier highlights the importance of the intensity of viral replication. A subset of those highly up-regulated proteins is also linked within a neurological disease network. This study serves as basis for future investigations to assess the prospective prognosis value of the CMV34 in larger cohorts. Moreover, these findings provide new insight on the pathobiology of CMV-induced fetal brain lesions and they open the perspective of new therapeutic options.

## Material and Methods

### Patients

Forty-seven amniotic fluid samples collected in the second trimester of pregnancy with CMV infected fetuses were extracted from the Necker Virology laboratory database and analyzed retrospectively. Prenatal data were reviewed, including fetal ultrasound and MRI examination and follow-up. Outcome was assessed by targeted neonatal examination or postmortem examination following termination of pregnancy (TOP). All infected fetuses were followed-up in the Fetal Medicine Unit at Necker Hospital using the same management. Amniocentesis was performed either because of suggestive fetal ultrasound symptoms or in the context of documented maternal primary infection. Fetal blood sample by cordocentesis under continuous ultrasound guidance is part of the management proposed in all infected cases. Fetal blood viral DNA-load and fetal platelet count are measured to be used as part of the prognostic assessment of all infected fetuses in the Fetal Medicine Unit at Necker Hospital. Cerebral MRI was performed after 30–32 weeks of pregnancy in all cases that were not terminated.

Amniotic fluid samples from women carrying a CMV infected fetus were divided into two cohorts. The discovery cohort was composed of 26 amniotic fluid samples obtained from infected fetuses that led to either termination of pregnancy (TOP) for severe brain lesions or symptomatic neonates (n = 13) or to asymptomatic neonates (n = 13). A second blinded cohort was used for validation and was composed of 21 amniotic fluid samples obtained from infected fetuses that led to TOP or to symptomatic neonates (n = 9) or from asymptomatic neonates (n = 12).

### Ethics

All women undergoing amniocentesis in the Fetal Medicine Unit at Necker Hospital gave written informed consent for CMV prenatal diagnosis to be performed on amniotic fluid and for the amniotic fluid sample left-over to be used for research purposes. According to French laws, an ethics statement from an Institutional Review board was not required for this work. Moreover, all women gave written informed consent for the use of clinical data as they were included in different clinical studies on congenital CMV in the Fetal Medicine Unit at Necker Hospital. These studies were approved by the Institutional Review Board of University Paris Ouest (IRB N°2011-001610-34 and 2013-A213-42).

### Classification between symptomatic and asymptomatic infection

Fetuses were classified as severely symptomatic when severe brain anomalies were identified by ultrasound, including ventriculomegaly measured ≥ 15mm, periventricular hyperechogenicity, hydrocephaly, microcephaly < -2DS, increased cisterna magna ≥ 8mm, vermian hypoplasia, porencephaly, lissencephaly, periventricular cysts or agenesis of the corpus callosum. In cases of TOP, severity was confirmed if postmortem examination showed microcephaly < 4SD, ventriculomegaly, white matter necrosis, associated with diffuse lesions of vasculitis and of encephalitis. Indeed, it has been documented in the literature that 77% of infected newborns with severe abnormalities on neonatal CT scan (intracranial calcifications, ventricular dilatation, white-matter abnormalities, cortical atrophy and migration abnormalities) develop at least one psychomotor sequela [39]. In another study 100% of newborns with microcephaly had severe mental retardation [40]. Therefore, severely abnormal brain imaging both on ultrasound and on MRI in utero is likely to be found mainly in cases destined to become neurologically symptomatic neonates.

Symptomatic or asymptomatic status at birth was evaluated by clinical examination, biological assessment (platelet count, liver enzymes, and bilirubin serum levels) and audiometric assessment by automated auditory brainstem response (AABR). Neonates with normal clinical examination, normal biological assessment and normal hearing were considered asymptomatic. Neonates with abnormal clinical examination and/or abnormal biological assessment, including petechiae, microcephaly, seizures, lethargy/hypotonia, poor suck, hepatosplenomegaly, thrombocytopenia and hearing loss were considered as symptomatic. Infected children are routinely followed-up with serial clinical examination and audiometric evaluation (AABR) performed at 4, 12, 18, 24 and 36 months of age.

For the purpose of the study, cases were classified into 3 groups. Cases with severe brain anomalies identified either at prenatal ultrasound or MRI, at necropsy or at birth were classified as severely symptomatic. Cases with isolated unilateral hearing loss > 40 decibels and/or vestibular syndrome identified at birth or at last follow-up visit were classified as moderately symptomatic. Asymptomatic cases at birth or at last available follow-up visit were classified as asymptomatic.

### CMV DNA quantification in fetal blood and in amniotic fluid

CMV DNA was extracted from 200 μl of amniotic fluid with the MagNaPure LC using the Total Nucleic Acid extraction kit (Roche Diagnostic, Meylan, France) and from 200μl of fetal whole blood using the QiaAmp DNA mini blood kit (Qiagen, Courtaboeuf, France). CMV DNA was amplified by CMV-R Gene (Argene BioMerieux, France) a real time commercial quantitative CMV PCR assay.

### Sample preparation, CE-MS and data processing

We have recently developed a sample preparation protocol for the analysis of the fetal urinary peptidome by CE-MS starting from 150 μl of fetal urine [41]. This protocol was also used for amniotic fluid. Briefly, immediately before preparation, amniotic fluid aliquots kept at –80°C were thawed and 150 μl aliquots were diluted with the same volume of 2 M urea, 10 mM NH4OH containing 0.2% SDS. Subsequently, samples were passed over a Centristat 20-kDa cut-off centrifugal filter device (Sartorius) in order to eliminate high molecular weight compounds. The filtrate was desalted using a NAP-5 gel filtration column (GE Healthcare) to remove urea and electrolytes. Lyophilisation of the sample was performed using a Savant speedvac SVC100H connected to a Virtis 3L Sentry freeze dryer (Fisher Scientific) and stored at 4°C until use. Shortly before CE-MS analysis, the samples were re-suspended in 10μL of HPLC grade H_2_O.

CE-MS analyses were performed as previously described [41–43] using a Beckman Coulter Proteome Lab PA800 capillary electrophoresis system (Beckman Coulter) on-line coupled to a micrOTOF II MS (Bruker Daltonic). The electro-ionization sprayer (Agilent Technologies) was grounded, and the ion spray interface potential was set between –4 and –4.5 kV. Data and MS acquisition methods were automatically controlled by the CE via contact-close-relays. Spectra were accumulated every 3 s, over a range of m/z 350 to 3000. Mass spectral ion peaks representing identical molecules at different charge states were deconvoluted into single masses using MosaiquesVisu software [44]. The software automatically examined all mass spectra from a CE-MS analysis for signals with a signal-to-noise ratio of at least 4 present in 3 consecutive spectra. Furthermore, the isotopic distribution was assessed, and charge was assigned based on the isotopic distribution, as well as conjugated masses, using a probabilistic clustering algorithm. This operation resulted in a list wherein all signals that could be interpreted are defined by mass/charge, charge, migration time, and signal intensity (ion counts). Time-of-flight mass spectrometry (TOF-MS) data were calibrated utilizing Fourier transform ion cyclotron resonance mass spectrometry (FT-ICR-MS) data as reference masses applying linear regression. Both CE-migration time and ion signal intensity (amplitude) showed high variability, mostly due to different amounts of salt and peptides in the sample. Normalization of the amplitude of the amniotic fluid peptides was based on sequenced endogenous “housekeeping” peptides (Table 6) that varied little among the samples. Based on the sequences most of the housekeeping peptides found in amniotic fluid were similar to those observed in urine, therefore allowing the application of a CE-MS procedure for sample analysis and data processing similar to the procedure used for urine [42,45].

**Table 6:** Amniotic fluid peptides used for normalization and their correspondence with postnatal urinary peptides used for normalization.

CE-MS housekeeping peptides from postnatal urine samples					LTQ-Orbitrap XL sequencing of amniotic fluid peptides	
Peptide ID	Mass (Da)	Mass SD	Sequence information*	Parent protein	Sequence information**	Mass (Da)***
**13342**	1016.45	0.02	ApGDKGESGPS	Collagen alpha-1 (I) chain		
**24117**	1194.55	0.01	SpGPDGKTGPPGp	Collagen alpha-1 (I) chain		
**27517**	1250.56	0.01	ApGDRGEpGPpGP	Collagen alpha-1 (I) chain		
**28747**	1268.57	0.01	SpGERGETGPpGP	Collagen alpha-1 (III) chain		
**35339**	1378.61	0.01	ApGEDGRpGPpGPQ	Collagen alpha-1 (II) chain		
**38605**	1435.66	0.01	SpGSPGPDGKTGPpGP	Collagen alpha-1 (I) chain		
**40243**	1451.66	0.01	SpGSpGPDGKTGPPGp	Collagen alpha-1 (I) chain		
**43543**	1508.68	0.02	GSpGSpGPDGKTGPPGp	Collagen alpha-1 (I) chain		
**44618**	1523.74	0.02	GDPGPPGpPGpPGpPAI	Collagen alpha-1 (XV) chain	GDPGPPG*PP*Gp*P*G*P*PAI [3]	1523.73
**48106**	1579.71	0.01	SpGSpGPDGKTGPPGpAG	Collagen alpha-1 (I) chain		
**57531**	1737.78	0.02	TGSpGSpGPDGKTGPPGpAG	Collagen alpha-1 (I) chain	TGSpGSpGPDGKTGPPGpAG [3]	1737.78
**61945**	1834.83	0.02	GLpGTGGPpGENGKpGEPGp	Collagen alpha-1 (III) chain		
**71312**	2025.87	0.02	SEGSpGHpGQpGpPGPPGApGp	Collagen alpha-1 (III) chain	SEGSpGHpGQpGp*P*GPPGA*P*Gp [6]	2025.87
**72533**	2046.92	0.03	PpGEAGKpGEQGVpGDLGApGP	Collagen alpha-1 (I) chain		
**73246**	2063.93	0.02	NGDDGEAGKpGRpGERGPpGP	Collagen alpha-1 (I) chain	NGDDGEAGKpGRPGERG*PP*G*P* [3]	2063.93
**74065**	2078.93	0.01	DAGApGApGGKGDAGApGERGPpG	Collagen alpha-1 (III) chain	DAGApGApGGKGDAGApGERGPpG [4]	2078.93
**74902**	2087.97	0.02	GppGEAGKPGEQGVPGDLGAPGp	Collagen alpha-1 (I) chain	G*P*pGEAGK*P*GEQGV*P*GDLGAPG*P* [3]	2087.98
**75846**	2103.96	0.02	GPpGEAGKpGEQGVpGDLGApGP	Collagen alpha-1 (I) chain	GPpGEAGKpGEQGVpGDLGA*P*G*P* [4]	2103.98
**78332**	2159.00	0.03	AGPpGEAGKpGEQGVpGDLGAPGP	Collagen alpha-1 (I) chain	AG*P*pGEAGK*P*GEQGV*P*GDLGAPG*P* [3]	2159.02
**78843**	2169.98	0.02	NSGEpGApGSKGDTGAKGEpGPVG	Collagen alpha-1 (I) chain	NSGEpGApGSKGDTGAKGEpGPVG [3]	2169.98
**79136**	2175.01	0.02	AGPpGEAGKpGEQGVpGDLGApGP	Collagen alpha-1 (I) chain	AGPpGEAGKpGEQGVpGDLGApGP [4]	2175.01
**81758**	2220.99	0.02	ADGQpGAKGEpGDAGAKGDAGPpGP	Collagen alpha-1 (I) chain	ADGQpGAKGEpGDAGAKGDAGPpGP [3]	2220.99
**82026**	2226.99	0.01	GNSGEpGApGSKGDTGAKGEpGPVG	Collagen alpha-1 (I) chain		
**85761**	2292.02	0.02	ADGQpGAKGEpGDAGAKGDAGPpGPA	Collagen alpha-1 (I) chain	ADGQpGAKGEpGDAGAKGDAGPpGPA [3]	2292.03
**98660**	2564.15	0.03	GApGQNGEpGGKGERGApGEKGEGGPpG	Collagen alpha-1 (III) chain	GApGQNGEpGGKGERGApGEKGEGGPpG [4]	2564.15
**104786**	2679.20	0.03	NRGERGSEGSPGHpGQpGppGpPGAPGP	Collagen alpha-1 (III) chain		
**105352**	2695.20	0.02	NRGERGSEGSpGHpGQpGppGPPGAPGp	Collagen alpha-1 (III) chain	NRGERGSEGS*P*GHpGQpGppG*PP*GAPG*P* [6]	2695.20
**107460**	2742.25	0.03	KNGETGPQGPpGPTGPGGDKGDTGPpGPQG	Collagen alpha-1 (III) chain		
**111001**	2825.27	0.03	ERGEAGIpGVpGAKGEDGKDGSpGEpGANG	Collagen alpha-1 (III) chain	ERGEAGIpGVpGAKGEDGKDGSpGEpGANG [4]	2825.27

* Lower case p indicated hydroxyproline.

** Lower case p indicated unambiguous hydroxylated proline sites and at the same proline residues than sequence obtained from postnatal urine. Hydroxylated proline sites underlined and in italic indicated different possible site. The total number of hydroxyproline are indicated in square brackets.

*** Experimental mass (Da) are obtained from the mean of all MS/MS data for this species on a LTQ-Orbitrap XL with a mass accuracy <4 ppm.

### Statistical analysis and biomarker identification

Normalized levels of amniotic fluid peptides were compared between symptomatic and asymptomatic patients’ groups for the identification of amniotic fluid proteome biomarkers. Only peptides with a P-value<0.05 corrected for multiple testing (Benjamini- Hochberg) were considered significant [46]. The number of peptides with differential abundance was reduced to a support vector machine (SVM) classifier with 34 peptides (CMV34) by a take-one-out procedure that had similar performance for the classification of the patients in the discovery CMV patient cohort. Sensitivity and specificity of the previously defined biomarker classifiers, and 95% confidence intervals (95% CI) were calculated using receiver operating characteristic (ROC) plots (MedCalc version 14.8.1, MedCalc Software, Belgium).

### Sequencing of peptide biomarkers

Candidate biomarkers and other native peptides from fetal urine and amniotic fluid were sequenced using LC-MS/MS and CE-MS/MS analysis [47]. LC-MS/MS analysis experiments were performed on a Dionex Ultimate 3000 RSLC nano flow system (Dionex, Camberly UK). For CE-MS/MS, the samples were injected under constant flow and pressure conditions at a pH of 2.2 to ensure that all peptides are positively charged. Both, CE and LC, were directly interfaced with an LTQ-Orbitrap XL (Thermo Finnigan, Bremen, Germany), using data-dependent HCD MS/MS sequencing of a maximum of the top 20 ions. All resultant MS/MS data were analysed using Proteome Discoverer 1.2 (activation type: HCD; min-max precursor mass: 790–6000; precursor mass tolerance: 10 ppm; fragment mass tolerance: 0.05 Da; S/N threshold: 1) and were searched against the Uniprot human non-redundant database without enzyme specificity. No fixed modifications were selected, oxidation of methionine and proline and deamidation of aspartic acid and glutamine were selected as variable modifications. The peptide data were extracted using high confidence peptides, defined by an Xcorr≥1.9, a delta mass between experimental and theoretical mass ± 5 ppm, absence of cysteine in the sequence as without reduction and alkylation it is forms disulphide bonds, absence of oxidized proline in protein precursors other than collagens or elastin, and top one peptide rank filters.

For further validation of peptide identification, the strict correlation between peptide charge at pH 2 and CE-migration time was utilized to minimize false-positive identification rates [48]. Calculated CE-migration time of the sequence candidate based on its peptide sequence (number of basic amino acids) was compared to the experimental migration time. Peptides were accepted only if they had a mass deviation below ±50 ppm and a CE-migration time deviations below ±2 min.

### Ingenuity pathway analysis

Ingenuity Pathway Analysis software (version 24390178, last release 18/06/2015, www.ingenuity.com) was performed using the parent proteins peptides with differential abundance in the symptomatic CMV infections. As urine, amniotic fluid is very rich in different collagen fragments. Therefore, collagens were omitted from the Ingenuity pathway analysis to avoid a bias in the analysis towards fibrosis and conjunctive tissue remodeling. All Ingenuity output related to cancer was omitted.

## Supporting Information

S1 Table Patient data of the severely symptomatic and asymptomatic fetuses from the discovery and validation cohorts. (XLS)

S2 Table Patient data of the moderately symptomatic fetuses from the additional cohort. (XLS)

S1 Dataset Peptidome analysis of the asymptomatic fetuses from the discovery cohort. (XLS)

S2 Dataset Peptidome analysis of the symptomatic fetuses from the discovery cohort. (XLS)

S3 Dataset Peptidome analysis of the asymptomatic fetuses from the validation cohort. (XLS)

S4 Dataset Peptidome analysis of the symptomatic fetuses from the validation cohort. (XLS)

S1 Fig Correlation analysis of the beta-2-microglobulin fragment (ID 44679) and CMV DNA levels in amniotic fluid (A) and fetal blood (B). (TIF)
