# Supplementary figures and images for: Identification of Symptomatic Fetuses Infected with Cytomegalovirus Using Amniotic Fluid Peptide Biomarkers
Source: PLoS Pathog. 2016 Jan 25;12(1):e1005395. doi: 10.1371/journal.ppat.1005395 (PMC4726449; doi:10.1371/journal.ppat.1005395)

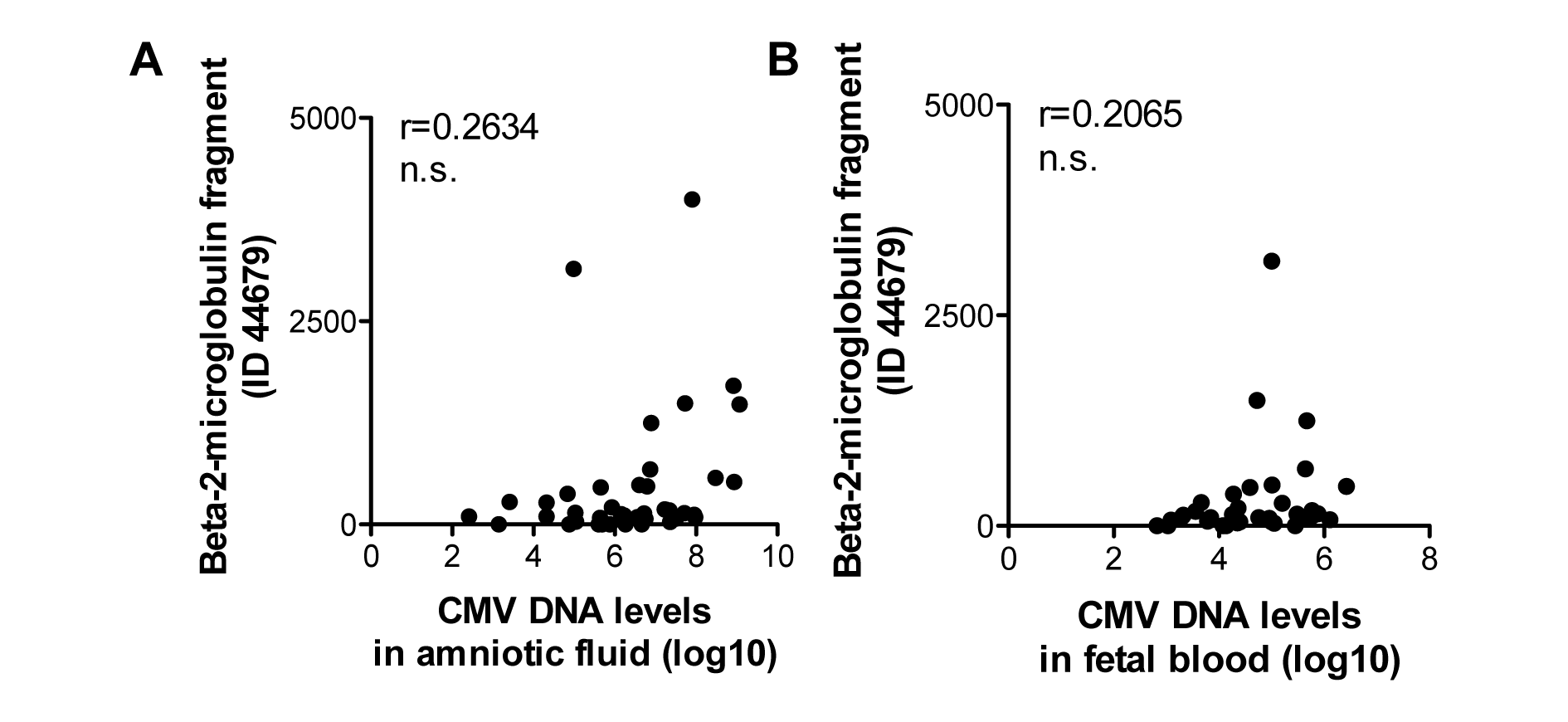

Supplement: S1 Fig — (TIF) [file ppat.1005395.s007.tif]
